# The Impact of High-Flow Nasal Cannula Therapy on Acute Respiratory Distress Syndrome Patients: A Systematic Review

**DOI:** 10.7759/cureus.41219

**Published:** 2023-06-30

**Authors:** Ahmed M Abdelbaky, Wael G Elmasry, Ahmed H. Awad, Sarrosh Khan, Maryam Jarrahi

**Affiliations:** 1 Intensive Care Unit, Dubai Academic Health Corporation - Rashid Hospital, Dubai, ARE; 2 Internal Medicine, Dubai Academic Health Corporation - Rashid Hospital, Dubai, ARE

**Keywords:** acute respiratory distress syndrome, ards, covid-19, high flow nasal cannula (hfnc), hypoxia, non-invasive ventilation (niv)

## Abstract

High-flow nasal cannula (HFNC) is a novel oxygenation approach in the management of acute respiratory distress syndrome (ARDS). This systematic review was focused on evaluating current evidence concerning the efficacy of HFNC in ARDS and its comparison with standard treatment approaches. For this review, a systematic search was undertaken in PubMed, Cumulative Index to Nursing and Allied Health Literature (CINAHL), Embase, Web of Science, Cochrane Library, and Google Scholar to identify relevant studies. Preferred Reporting Items for Systematic Reviews and Meta-Analyses (PRISMA) guidelines were followed. All those studies that investigated the impact of HFNC on ARDS patients and were published in the English language were included. The literature search from all databases provided 6157 potentially relevant articles from PubMed (n = 1105), CINAHL (n = 808), Web of Science (n = 811), Embase (n = 2503), Cochrane database (n = 930), and Google Scholar (n = 46). After the exclusion of studies that did not fulfill the criteria, 18 studies were shortlisted for the scope of this systematic review. Among the included studies, five focused on HFNC's impact on COVID-19-related ARDS, whereas 13 studies focused on HFNC's impact on ARDS patients. Most studies demonstrated the efficacy of HFNC in managing ARDS, with some studies showing comparable efficacy and higher safety compared to noninvasive ventilation (NIV). This systematic review highlights the potential benefits of HFNC in ARDS management. The findings show that HFNC is effective in reducing the respiratory distress symptoms, the incidence of invasive ventilation, and the adverse events associated with ARDS. These findings can help clinical decision-making processes and contribute to the evidence base for optimal ARDS management strategies.

## Introduction and background

Acute respiratory distress syndrome (ARDS) is a severe form of acute respiratory failure characterized by hypoxemia, dyspnea, and pulmonary infiltrates. ARDS can be defined as a life-threatening condition characterized by poor oxygenation and non-compliant or "stiff" lungs. This condition is often linked with capillary endothelial and alveolar damage. Most of the cases of ARDS are moderate to severe, accounting for 75%, whereas only 25% are mild in nature [1]. Berlin criteria of diagnosis are widely accepted for the classification of ARDS based on PaO_2_/FiO_2_, with 201-300 mmHg being classified as mild ARDS, 101-200 mmHg as moderate, and less than 100 mmHg as severe ARDS [2]. In recent decades, significant improvements have been observed in critical care management. However, ARDS remains a significant concern for global morbidity and mortality [3]. Oxygenation therapy is critical in the management of ARDS. Traditional oxygenation approaches like face masks and nasal prongs have certain limitations like poor oxygenation and discomfort to patients that hinder their applicability. Due to this gap, noninvasive ventilation (NIV) and high-flow nasal cannula (HFNC) oxygen therapy are widely adopted to provide higher inspired oxygen fractions (FiO_2_). NIV is utilized as an alternative option to invasive mechanical ventilation, especially in cases of acute-on-chronic respiratory failure. However, the use of NIV in cases of ARDS has had mixed results [3,4]. Despite its advantages, NIV has been implicated with several drawbacks such as intolerance and discomfort in patients. These adverse consequences of NIV have been associated with treatment discontinuation in roughly 22% of the patients [5]. Additionally, NIV is not considered a viable option in patients with neurological disorders, which leaves conventional oxygenation treatment for such patients. However, conventional oxygen therapy has its limitations including lack of flow, limited FiO_2_, intolerance, and claustrophobia owing to the application of face masks [6].

HFNC has emerged as a novel approach that has shown promising results in counteracting the potential drawbacks of conventional oxygenation therapies. HFNC delivers high-flow oxygen, which provides several advantages over traditional oxygen delivery methods [7]. HFNC can deliver higher levels of oxygenation, improve ventilation and mucociliary clearance, and provide greater patient comfort and tolerance. HFNC delivers heated and humidified oxygen at a high flow of 50-60 L/min via a wide-bore nasal cannula [8]. The utilization of a high flow of oxygen facilitates the observation of enhanced inspiratory flow amplifications in individuals suffering from hypoxemia, thereby minimizing the dilution of oxygen and ensuring the delivery of an inspired oxygen fraction (FiO_2_) that closely approximates the predetermined FiO_2_ value [9]. A significant benefit conferred by HFNC therapy is its ability to sustain adequate oxygenation throughout the apneic phase following the administration of anesthesia, which effectively circumvents hypoxemia. NIV, on the other hand, is removed at this phase [10]. A prospective study reported that HNFC reduced hypoxemia incidence compared to standard oxygen during the process of laryngoscopic intubation for patients in immediate need of mechanical ventilation [11]. However, subsequent studies did not validate these findings [12,13].

Several physiological benefits have been identified in HFNC therapy including lower inspiratory resistance, diminished dyspnea, and less effort for breathing in patients [14]. HFNC treatment has also demonstrated sustained effects in patients with ARDS [15,16]. Despite the favorable findings reported in these studies, the extent to which this technique can be applied and its generalizability remain uncertain due to certain limitations. These include the restricted focus on the immediate effects of HFNC in a short-term context, low sample size, and selection bias in these studies. Furthermore, indications for HFNC are not as clearly defined as they are for NIV, with a wide range of suggested applications ranging from palliative care situations to pulmonary infections or cardiac failure [17,18]. Several studies have investigated the effectiveness of HFNC in ARDS patients, with varying results. The potential benefits of HFNC therapy in ARDS have generated significant interest among clinicians and researchers, which has led to a growing body of literature on the topic. This review aims to provide a comprehensive analysis of the available evidence on the impact of HFNC therapy on ARDS patients. Specifically, this review will examine the effects of HFNC therapy on oxygenation, respiratory rate, rates of intubation, and mortality in ARDS patients. The review will also explore potential mechanisms of action underlying the beneficial effects of HFNC therapy as well as the limitations and challenges associated with its use in ARDS patients.

## Review

Methods

The protocols for the current systemic were devised in adherence to the guidelines prescribed by the Preferred Reporting Items for Systematic Reviews and Meta-Analyses (PRISMA) [19].

Search strategy and data sources

For this review, comprehensive research was conducted in several databases to find relevant studies that explored the impact of HFNC oxygen therapy in ARDS patients. We ran separate searches in PubMed, Web of Science, Cumulative Index to Nursing and Allied Health Literature (CINAHL), Embase, and Cochrane Library databases to find relevant studies. The research was undertaken using a combination of different keywords including “high flow nasal cannula oxygen therapy,” “high flow nasal oxygen,” or “high flow nasal cannula oxygenation” and “respiratory distress syndrome” or “ARDS” (Appendix 1). Related terms, alternatives, and plurals were also considered. Further, we also search Google Scholar and reference sections of the selected studies to increase the body of evidence in this systemic review. We included studies that met the following criteria: (1) studies that included ADRS patients, (2) studies that investigated the impact of HFNC oxygen therapy in ARDS patients, and (3) studies that were published in the English language. Studies that did not assess oxygenation therapy in ARDS were excluded from the scope of this review.

Data collection process

All the matched articles from database searches were transferred to the reference manager (EndNote 20, Thomson Reuters) with the exclusion of duplicate and non-English titles. After that, the endnote file was transferred to Rayyan, a web-based software to expedite the initial screening of the search results [20]. The further process was divided into three stages: (1) the selection of studies based on title and abstract that were eligible for inclusion in the review, (2) a thorough analysis of the eligible articles keeping in view the aim of the review, and (3) further search was refined based on the exclusion and inclusion criteria, and data were obtained in the form of notes regarding the intervention used in studies, the number of participants, and the methods used.

Flow diagram

**Figure 1:**
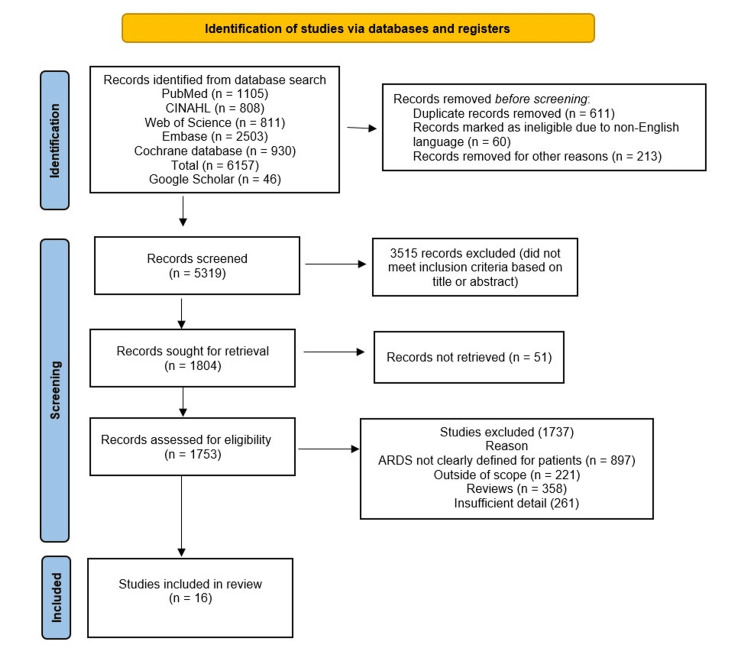
PRISMA flow diagram of the systemic review

The study design adheres to the PRISMA flow diagram and protocol [21], which outlines the systematic approach from identifying relevant articles to selecting articles that meet the eligibility criteria for further analysis (Figure 1).

Results

Included Studies

The literature search from all databases provided 6157 potentially relevant articles from PubMed (n = 1105), CINAHL (n = 808), Web of Science (n = 811), Embase (n = 2503), and Cochrane database (n = 930). Additionally, 46 studies were identified from Google Scholar searches. After the exclusion of duplicate studies and non-English publications, only 5319 records were further analyzed. Based on keywords and abstracts, 3515 publications were removed from the scope of this review. Of the residual corpus of literature, a thorough assessment was performed to identify the 16 most relevant studies for inclusion in the scope of the current review.

Study Characteristics

Out of the 16 studies reviewed, 10 studies investigated HFNC in COVID-19-related ARDS. The remaining six studies assessed the efficacy of HFNC in ARDS patients due to various conditions.

Studies Investigating HFNC in COVID-19-Related ARDS

In the current review, most studies manifested the efficacy of HFNC in the management of ARDS. All studies that investigated COVID-19-related ARDS were retrospective. Nine studies showed that HFNC reduced respiratory complications and was associated with low ARDS-related adverse events, whereas one study showed similar efficacy of noninvasive positive pressure ventilation (NIPPV) and HFNC treatment (Table 1). Only four studies defined ARDS according to Berlin's criteria of diagnosis [22-25]. A total of 1662 participants with ARDS were included in COVID-19-related studies, out of which only 290 participants were clearly described as moderate to severe ARDS, whereas 17 patients were characterized as severe ARDS. The remaining 1355 participants were described as COVID-19-related ARDS patients only.

**Table 1:** Summary of studies investigating HFNC in COVID-19-related ARDS ARDS: Acute respiratory distress syndrome; HFNC: High-flow nasal cannula; SpO_2_, Saturation of peripheral oxygen; PaO_2_: Supranormal arterial oxygen; DNIO: Do not intubate order; ICU: Intensive care unit; ROX index: The ratio of oxygen saturation as measured by pulse oximetry/FiO_2_ to respiratory rate; CPAP: Continuous positive airway pressure; NIPPV: Noninvasive positive pressure ventilation; NIV: Noninvasive ventilation; IMV: Invasive mechanical ventilation.

Author	Study design	Participants	Intervention	Key findings	Conclusion
Panadero et al. [22]	Retrospective study	40 ARDS patients	HFNC	The mortality rate was 22.5%. At day 30, the intubation rate was 52.5%. The SpO_2_/FiO_2_ ratio was better in patients not requiring intonation.	HFNC is useful for the management of ARDS.
Delbove et al. [3]	Retrospective study	46 ARDS	HFNC-DNIO HFNC-intubated	The PaO_2_/FiO_2_ ratio in the HFNC-DNIO group was 109, and the associated hospital mortality rate was 54.5%. Patients in the HFNC-intubation group exhibited worse respiratory rates during their ICU stay compared to those in the HFNC-only group (37 versus 33 minutes, p < 0.05) as well as a more pronounced decline in their ICU admission PaO_2_/FiO_2_ ratios (121 versus 191, p < 0.001).	HFNC is a good option for COVID-19-related ARDS.
Alay et al. [23]	Retrospective study	22 ARDS patients	HFNC	Only 2 patients required intubation. The duration of HFNC was 4.8 ± 3.6 days, and the length of ICU stay was 17.4 ± 6 days. The overall mortality was 13.6%.	HFNC reduces the risk of reintubation.
Sekar et al. [26]	Retrospective study	127 patients	HFNC (75%), CPAP (25%)	26% of subjects had a failure and required invasive mechanical ventilation.	ROX index of <2.97 at 2 hours and <3.63 at 6 hours predicted failure.
Zhao et al. [27]	Retrospective study	41 patients	NIPPV group and HFNC group	Complications in the HFNC group (41.2%) were lower compared to NIPPV (87.5%), and the need for purification of blood was also lower (0% vs. 29.4%, p < 0.05). Mortality (58.3% vs. 52.9%) and intubation rate (66.7% vs 70.6%) were comparable between NIPPV and HFNC groups.	HFNC is similar to NIPPV in ARDS patients.
Fulya et al. [24]	Retrospective study	17 patients	HFNC	Patients intubated within 12-24 h had lower mortality compared to >24 h.	The cutoff value for the ROX index was 2.84 for the need for intubation.
Kerai et al. [28]	Retrospective study	85 patients with moderate to severe ARDS	HFNC	The success rate with HFNC was 48.2%. ROX indices post-initiation was significantly higher in the successful group (p ≤ 0.001).	Baseline oxygen saturation impacts the outcome of HFNC.
Carpagnano et al. [25]	Retrospective study	78 patients with moderate to severe ARDS	HFNC, NIV	28.6% of patients treated with HFNO died, and 41.0% of patients treated with NIV died.	HFNC reduces the risk of mortality in ARDS patients.
Procopio et al. [29]	Retrospective study	5 patients	HFNC, CPAP, NIV	HFNC treatment resulted in a PaO_2_ increase (48.80 vs.71.98 mm Hg). Moreover, PaO_2_/FiO_2_ ratio increased from 231.8 to 342.4 mm Hg; p = 0.06).	HFNC is better than CPAP.
Jog et al. [30]	Retrospective study	1,201 patients with PaO_2_/FiO_2_ ratio < 150	HFNC, NIV, and invasive mechanical ventilation (IMV)	The need for IMV was significantly lower in the HFNC group (48.3%) compared to NIV (61.6%) (p < 0.001). The mortality assessment after 28 days showed significantly decreased risk in HFNC (44.9%) compared to NIV (59.9%) (p < 0.001).	HFNC had better outcomes compared to NIV.

In patients that received HFNC, the mean rate of re-intubation was 46.11% in COVID-19-related ARDS patients. In HFNC-treated ARDS patients, the mean mortality rate was 25.01%. Five studies described the ratio of oxygen saturation (ROX) index to evaluate the success of HFNC. Changes in PaO_2_/FiO_2_ (P/F), reintubation required, percentage mortality, ROX index, and cut-off value are described in Table 2.

**Table 2:** Change in PaO2/FiO2 (P/F), reintubation required, percentage mortality, ROX index, and cutoff value in COVID-19-related ARDS patients HFNC: High-flow nasal cannula; SpO_2_: Saturation of peripheral oxygen; PaO_2_: Supranormal arterial oxygen; ROX index: The ratio of oxygen saturation as measured by pulse oximetry/FIO_2_ to respiratory rate; IMV: Invasive mechanical ventilation; S/F: SpO_2_/FiO_2_; P/F: PaO_2_/FiO_2_; N/A: Not attempted.

Author	Change in PaO_2_/FiO_2_ (P/F) or SpO_2_/FiO_2_ (S/F)	Reintubation required	Mortality	ROX index and its cutoff value
Panadero et al. [22]	S/F in non-intubated vs intubated (113.4 ± 6.6 vs 93.7 ± 6.7)	52.5%	22.5%	ROX indicator of intubation = 4.94
Delbove et al. [3]	After the intervention, the HFNC group improved from 191 to 248, and the intubated group improved from 121 to 207.	57%	54.5%	N/A
Alay et al. [23]	After HFNC, the group improved from 111.4 ± 31.9 to 180 ± 46.	4.5%	13.6%	N/A
Sekar et al. [26]	At 2 h, 109 vs 80 in HFNC successful and unsuccessful patients	26%	N/A	ROX at 2 h < 2.97; at 6 h < 3.63
Zhao et al. [27]	The S/F ratio decreased and RR increased in the HFNC group compared to the baseline.	58.3%	52.9%	N/A
Fulya et al. [24]	N/A	67.1%	4.34%	ROX indicator of intubation = 2.84
Kerai et al. [28]	P/F in the HFNC success group was 115, whereas it was 87 in the failure group.	51.8%	N/A	At 2, 6, and 12 post-HFNC, the ROX index was 5.43, 5.74, and 6.12, respectively.
Carpagnano et al. [25]	N/A	N/A	7.4%	N/A
Procopio et al. [29]	After HFNC, P/F increased from 231.8 to 342.4.	-	0%	At 2 and 12 post-HFNC, the ROX index was 7.42 and 9.3, respectively.
Jog et al. [30]	Discharged and not required IMV, the P/F value was 92.6 compared to P/F 81.0 in IMV patients.	51.7%	44.9%	N/A

Studies Investigating HFNC in ARDS Patients

In the current review, six studies assessed HFNC in ARDS patients (Table 3). Three studies included participants who were either infants or children below the age of 12 years, whereas the other three studies focused on ARDS in adult patients. A total of 307 participants were included in all studies, out of which only 16 were adequately classified as severe ARDS patients, 31 as moderate ARDS patients, and 37 as mild ARDS cases based on the Berlin definition of ARDS. The remaining patients were not classified as mild, moderate, or severe ARDS, rather the authors described them as ARDS or RDS cases. The mean reintubation rate after HFNC was 25.5% in ARDS patients. The mean mortality percentage was 5.66%. All studies reported that PaO_2_/FiO_2_ (P/F) was higher after HFNC from the baseline value. Furthermore, the lower P/F indicated a higher risk of HFNC failure. No study used the ROX index to determine the risk of HFNC failure.

**Table 3:** Summary of studies that investigated HFNC in ARDS patients ARDS: Acute respiratory distress syndrome; HFNC: High-flow nasal cannula; SpO_2_: Saturation of peripheral oxygen; PaO_2_: Supranormal arterial oxygen; ICU: Intensive care unit; CPAP: Continuous positive airway pressure; NIV: Noninvasive ventilation; N/A: Not attempted.

Author	Study design	Participants	Intervention	Reintubation rate	Change in PaO_2_/FiO_2_ (P/F)	Mortality %	Key findings	Conclusion
Sztrymf et al. [31]	Pilot prospective study	38 patients with persistent ARDS	HFNC	23.6%	From baseline of 102 at 1 h after HFNC to 169 and 187 after 24 h	0%	After 15 minutes of HFNC start, respiratory rate, heart rate, dyspnea score, supraclavicular retraction, and thoracoabdominal asynchrony were significantly reduced.	HFNC is a good option for oxygenation in ICU patients with acute respiratory failure.
Messika et al. [32]	Observational study	45 ARDS patients	HFNC	40%	In the successful HFNC group, P/F was 145.3 compared to 115.3 in re-intubated (base value = 137)	11%	Pneumonia was present in 82% of ARDS patients. 40% of patients were intubated. SAPS II score, organ failure, and higher breathing frequency after HFNC were predictors of HFNC.	HFNC is a good choice as the first line of treatment in ARDS patients.
Frat et al. [33]	Prospective study	23 patients with ARDS	HFNC NIV	35%	The P/F difference between non-intubated and intubated was 122 and 128, respectively.	20%	PaO_2_ increased from 83 mmHg to 108 mmHg and 125 mmHg in HFNC and NIV compared to standard oxygen therapy (p < 0.01). HFNC was better tolerated compared to NIV.	HFNC allows improvement in tachypnea and oxygenation in ARDS.
Spentzas et al. [34]	Observational study	46 children	HFNC	10.8%	N/A	0%	A significant improvement was seen in the modified COMFORT score, oxygen saturation, and respiratory clinical scale.	HFNC improved clinical outcomes in patients.
Shoemaker et al. [35]	Retrospective study	101 infants	CPAP, HFNC	18%	N/A	3%	95% of infants received HFNC compared to 12% in CPAP. There was no difference in ventilator days, mortality, and blood infections in both groups.	HFNC is well-tolerated by premature infants.
Kadivar et al. [36]	Randomized clinical trial	54 preterm infants	HFNC, CPAP	25.9%	N/A	0%	The rate of hospitalization and oxygenation requirement was not significant in both groups. However, the rate of intubation was higher in HFNC compared to CPAP.	HFNC and CPAP are comparable in terms of efficacy in preterm infants.

Discussion

This review shows that HFNC can improve oxygenation in ARDS patients and be associated with fewer adverse complications compared to other oxygenation approaches. The present review synthesized the recent evidence regarding the HFNC approach for oxygenation in ARDS patients. The review focused on synthesizing the quantitative data and did not perform qualitative data analysis as it was not the scope of the present review. The results showed that HFNC can reduce the incidence of intubation in COVID-related ARDS patients and reduce the risk of mortality. A retrospective study included in the review propagated that a ROX index below 4.94 serves as a predictor for the need for intubation [22]. ROX index is the quotient between pulse oximetry divided by the fraction of inspired oxygen and the respiratory rate. ROX index is a useful indicator to identify patients who are at low risk of HFNC failure and can continue therapy even after 12 hours. Some studies have suggested that the ROX index has the best prediction accuracy [22,37].

The most suitable cutoff point of the ROX index is considered 4.88 after 12 hours of HFNC and was associated with a low risk of mechanical ventilation. However, some studies have disagreed on whether the ROX index is the best predictor of HFNC failure. The main criticism centered around this is that the ROX index does not consider the most common clinical variable such as heart rate and PaO_2_/FIO_2_. An elevated heart rate can potentially serve as an indicator of heightened sympathetic activation attributable to augmented respiratory effort. A recent study demonstrated that a modified ROX index with the incorporation of HR can serve as a better predictor compared to the ROX index alone [38]. A new modified predictor of HFNC failure (Delta POX-HR) has been described by Kansal et al. by incorporating HR and substitution of PF ratio for SF ratio in addition to respiratory rate [39].

The study by Delbove et al. reported that almost 57% of patients were intubated after the initiation of HFNC therapy. Their findings showed that intubated patients had worse outcomes compared to the HFNC-only group. The respiratory rates per minute in ICU and ICU admission rates were worse compared to the HFNC-only group [3]. Previously, a meta-analysis of 17 studies showed that HFNC was not linked to a reduced need for intubation [40]. However, these findings were contradicted by Hamou et al. who reported that HFNC can be used as the first line of oxygenation support in ARDS patients and was associated with a low risk of intubation [41]. Delbove et al. also concluded that ARDS characterized by a PaO_2_/FiO_2_ below 150 and a respiratory rate exceeding 35 breaths per minute can be considered a prognostic indicator of the need for endotracheal intubation [3].

In contrast to HFNC therapy, both continuous positive airway pressure (CPAP) support and NIV pose discomfort to patients and necessitate substantial man-machine cooperation. The present review did not find any superiority of continuous positive airway pressure (CPAP) and NIV over HFNC. Zhou et al. reported that NIV did not reduce intubation rates in patients with COVID-19-related ARDS compared to HNFC [27]. Similarly, a retrospective study included in this review reported that HFNC can be used in patients who have a prior failure with CPAP and NIV [29]. Due to the better tolerability of HFNC, it has been used in between sessions of NIV to avoid oxygenation impairment. These findings have been shared by Frat et al. who concluded that HFNC improves oxygenation and tachypnea in ARDS patients [33].

Similarly, the efficacy of HFNC has been described in preterm infants as well. A study by Shoemaker et al. showed that there was no difference in adverse outcomes between HFNC and NCPAP [35]. A randomized clinical trial (RCT) by Kadivar et al. showed that HFNC was associated with a higher rate of intubation compared to CPAP [36]. Apart from preterm infants, HFNC has been shown to improve respiratory scale score, COMFORT scale, and oxygenation saturation [34].

An RCT by Coudroy et al. compared HFNC and NIV in immunocompromised patients [42]. Their findings showed that NIV and HFNC had no difference in mortality rate and other secondary outcomes. However, there was a great decrease in discomfort after HFNC compared to NIV. This point demonstrates the superiority of HFNC over NIV as immunocompromised individuals bear a significant amount of pain and discomfort throughout the course of the disease. Another study evaluated prognostic factors for adverse outcomes and mortality in immunocompromised individuals treated with HNFC. Duration of HFNC before intubation has been described as the major factor for mortality along with disease severity. Furthermore, FiO_2_ at initiation and SpO_2_ after initiation of HFNC can be considered risk factors for intubation [43]. A significant amount of evidence has highlighted that HFNC can be used as the first line of therapy in ARDS patients who require oxygenation [32]. Although this systemic review has found that HFNC is a beneficial approach for oxygenation in ARDS patients, it can manifest some complications in treated subjects. So far, only a few studies have investigated the safety of HFNC. A study by Veiga et al. reported that out of 70 patients that were treated with HFNC therapy, 10% developed epistaxis during HFNC [44]. Similarly, Baudin et al. investigated the complication of HFNC therapy. Their findings showed that 0.6% of patients developed epistaxis under HFNC treatment, whereas 1% and 3% developed new pneumothoraces and chest tube-related air leaks [45]. A case series also reported three cases of severe barotrauma (two pneumothoraces and one pneumomediastinum) related to HFNC therapy [46]. However, the incidence of complications during HFNC is usually rare, with Baudin et al. reporting 0.9 complications per 100 HFNC treatment days [45]. In the present systemic review, we did not find any significant complications related to HFNC treatment.

## Conclusions

In conclusion, this systematic review found that HFNC is an efficacious approach to the management of ARDS. The findings of the included studies consistently demonstrated the efficacy of HFNC in the management of ARDS. In the context of COVID-19-related ARDS, the majority of studies showed that HFNC reduced respiratory complications and low rates of ARDS-related adverse events. In the broader context of ARDS, the reviewed studies collectively supported the use of HFNC as an effective intervention. The specific etiologies of ARDS varied among the studies, indicating that HFNC may be beneficial across different underlying conditions leading to ARDS. Overall, the evidence presented in this systematic review suggests that HFNC can be considered a viable treatment option for ARDS, including COVID-19-related ARDS. However, it is important to note that the majority of studies included in this review were observational or retrospective in nature, which may introduce bias and limit the strength of the conclusions. Further well-designed randomized controlled trials are needed to confirm these. In summary, HFNC shows promise as a potential therapeutic option for ARDS patients. Its noninvasive nature, ability to provide high-flow oxygenation, and favorable outcomes observed in the reviewed studies make it an attractive alternative to traditional oxygen therapy methods. Further research is warranted to optimize its implementation, determine the optimal patient selection criteria, and compare its effectiveness to other interventions for ARDS management.

## Appendices

Appendix 1

**Table 4:** Terms and strategy used for literature search MeSH: Medical Subject Headings; ARDS: Acute respiratory distress syndrome.

Terms and strategy used for literature search		
#1 High-flow nasal cannula oxygen therapy	#10 High-flow oxygen therapy	#19 Early acute respiratory distress syndrome
#2 High-flow nasal cannula oxygen	#11 High-flow therapy	#20 ARDS
#3 High-flow nasal cannula oxygen delivery	#12 Nasal high flow	#21 Acute lung injury
#4 High-flow nasal cannula supportive therapy	#13 OR/1-12	#22 Wet lung
#5 High-flow nasal cannula oxygenation	#14 Respiratory distress syndrome (MeSH)	#23 Non-cardiogenic pulmonary edema
#6 High-flow nasal cannula aerosol therapy	#15 Respiratory distress syndrome	#24 Hyaline membrane disease
#7 High-flow nasal cannula	#16 Acute respiratory distress syndrome	#25 Infant respiratory distress syndrome
#8 High-flow nasal therapy	#17 Adult respiratory distress syndrome	#26 OR/14-25
#9 High-flow nasal oxygen	#18 Acute respiratory distress syndrome ARDS	#27 13 AND 26
