# Correction: The Impact of High-Flow Nasal Cannula Therapy on Acute Respiratory Distress Syndrome Patients: A Systematic Review

**DOI:** 10.7759/cureus.c336

**Published:** 2025-10-01

**Authors:** Ahmed M Abdelbaky, Wael G Elmasry, Ahmed H. Awad, Sarrosh Khan, Maryam Jarrahi

**Affiliations:** 1 Intensive Care Unit, Dubai Academic Health Corporation - Rashid Hospital, Dubai, ARE; 2 Internal Medicine, Dubai Academic Health Corporation - Rashid Hospital, Dubai, ARE

This article has been corrected to fix the following typos in the PRISMA diagram:

- "Records assessed for eligibility" corrected from 1702 to 1753
- "Studies excluded" corrected from 1678 to 1737

